# Treatment Reality of Proximal Humeral Fractures in the Elderly—Trending Variants of Locking Plate Fixation in Germany

**DOI:** 10.3390/jcm12041440

**Published:** 2023-02-10

**Authors:** Robert Rischen, Jeanette Köppe, Josef Stolberg-Stolberg, Moritz Freistühler, Andreas Faldum, Michael J. Raschke, J. Christoph Katthagen

**Affiliations:** 1 Clinic for Radiology, University Hospital Muenster, Albert-Schweitzer-Campus 1, Building A1, 48149 Muenster, Germany; 2 Institute of Biostatistics and Clinical Research, University of Muenster, Schmeddingstrasse 56, 48149 Muenster, Germany; 3 Department of Trauma-, Hand- and Reconstructive Surgery, University Hospital Muenster, Albert-Schweitzer-Campus 1, Building W1, 48149 Muenster, Germany; 4 Medical Management Division—Medical Controlling, University Hospital Muenster, Niels-Stensen-Straße 8, 48149 Muenster, Germany

**Keywords:** health claims data, geriatric surgery, proximal humeral fracture, locking plate fixation, osteosynthesis, augmentation, cerclage, double plating, bone graft, cement

## Abstract

Background: The surgical treatment of proximal humeral fractures (PHFs) with locking plate fixation (LPF) in the elderly is associated with high complication rates, especially in osteoporotic bone. Variants of LPF such as additional cerclages, double plating, bone grafting and cement augmentation can be applied. The objective of the study was to describe the extent of their actual use and how this changed over time. Methods: Retrospective analysis of health claims data of the Federal Association of the Local Health Insurance Funds was performed, covering all patients aged 65 years and older, who had a coded diagnosis of PHF and were treated with LPF between 2010 and 2018. Differences between treatment variants were analyzed (explorative) via chi-squared or Kruskal–Wallis tests. Results: Of the 41,216 treated patients, 32,952 (80%) were treated with LPF only, 5572 (14%) received additional screws or plates, 1983 (5%) received additional augmentations and 709 (2%) received a combination of both. During the study period, relative changes were observed as follows: −35% for LPF only, +58% for LPF with additional fracture fixation and +25% for LPF with additional augmentation. Overall, the intra-hospital complication rate was 15% with differences between the treatment variants (LPF only 15%, LPF with additional fracture fixation 14%, LPF with additional augmentation 19%; *p* < 0.001), and a 30-day mortality of 2%. Conclusions: Within an overall decrease of LPF by approximately one-third, there is both an absolute and relative increase of treatment variants. Collectively, they account for 20% of all coded LPFs, which might indicate more personalized treatment pathways. The leading variant was additional fracture fixation using cerclages.

## 1. Introduction

The proximal humeral fracture (PHF) is the third most common fracture, accounting for 6% of all fractures [1]. With an incidence of 288/100,000 per year in elderly people and a further increase with age, as many as 73% of all PHFs occur in elderly individuals [2,3,4]. The PHF is associated with significant morbidity, mortality and health care costs [5]. Due to the combination of an aging population and a high incidence in geriatric patients, the relevance of PHFs and their treatment will gain further significance.

While simple fractures can be treated non-surgically, more complex fractures, displaced or unstable fractures, are predominantly treated operatively. Various re-constructive procedures are available, including percutaneous fixation, plate fixation and intramedullary nailing [6,7,8]. Lately, there has been an increase in surgical treatment, with locking plate fixation (LPF) being the most frequently chosen procedure [9,10,11]. Despite considerable improvements, there is a high complication rate of up to 44% and a re-operation rate of 11% after LPF, especially in the elderly and those with osteoporosis [12,13]. The most frequent complications are a loss of reduction, screw cut-out and avascular necrosis [14,15].

**Figure 1:**
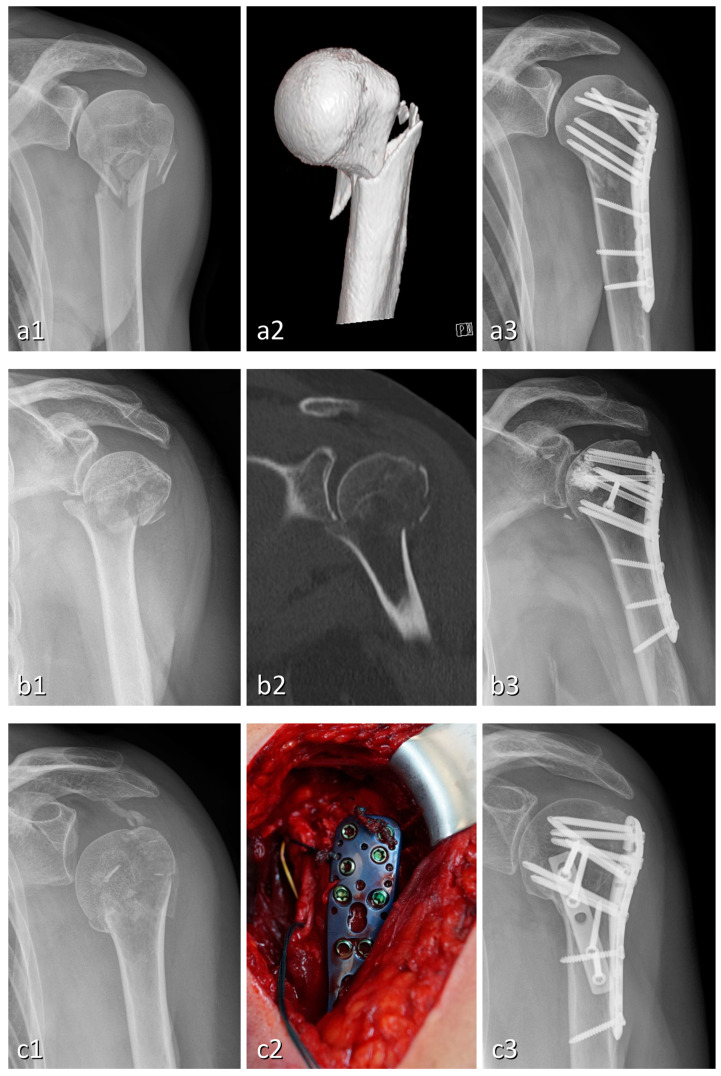
Radiographs of three cases of four-part proximal humeral fractures treated with different variants of locking plate fixation: PHILOS locking compression plate (**a**), plating with a combination of PHILOS locking compression plate, cement augmentation and additional screw (**b**), and double plating with one-third tubular plate (**c**). Each case is presented with pre- and post-operative X-ray (**a1**, **b1**, **c1** and **a3**, **b3**, **c3**, respectively) and an exemplary step in the diagnostic/therapeutic process: volume rendering technique (**a2**), CT with coronal re-construction (**b2**), surgery with plate implantation via deltoidopectoral approach (**c2**).

To address these complications and prevent failure of re-construction, several variants of LPF have been developed [16,17,18,19]. The placement of calcar screws in the inferomedial metaphysis improves medial support and reduces the risk of fixation failure [20]. Additional screws may be used to further increase stability [21]. Cerclages or sutures are predominantly used for fixation of the tuberosities to the plate [22]. In recent years, double plating has emerged (Figure 1), which adds another anterior, posterior or medial plate when the use of one lateral locking plate alone does not provide sufficient fixation in severely comminuted fractures [23,24]. Augmentation with autologous or allogenic bone grafts or synthetic composites, such as metals, ceramics and polymers, can significantly improve the bio-mechanical stability [25,26,27] as well as the clinical outcome in cases with poor bone quality, inadequate medial column support or high-risk fractures such as head-split or four-part fractures [28,29]. Bone augmentation for medial support enhancement or for metaphyseal void filling can be achieved with either an autograft (autologous) or allograft (allogenic); improved outcomes were reported for both fibular strut and cancellous bone from the iliac crest [30,31]. Cement augmentation encompasses different synthetic composites, such as calcium phosphate or polymethylmethacrylate, which can be injected as a liquid and harden in vivo [22]. It is mostly used for screw-tip augmentation (Figure 1) in order to increase the bone–implant interface and enhance implant anchorage in reduced bone stock [32,33].

There is still considerable uncertainty about the actual and desirable treatment variants of proximal humeral fractures. Most existing studies are based on clinical or bio-mechanical investigations or deal with LPF as a single entity without further sub-divisions. For example, trends across categories of surgical treatments of PHF were analyzed by Klug et al. [10] and Patel et al. [11]. However, a detailed analysis for LPF and its multiple variants in elderly patients is still missing. It can be assumed that care is provided inconsistently, since disagreement in science results in variations in care [9].

The main research questions were therefore as follows: Which procedures are actually performed and to what extent? Are there trends over time and across variants? Are there differences between variants regarding patient characteristics, outcome, duration of hospital stay and accrued cost?

The aim of this study was to evaluate the actual treatment reality beyond specialized and highly publishing centers and to provide a base for further optimization of LPF in clinical practice.

## 2. Materials and Methods

We conducted a retrospective study based on the health claims data of the Federal Association of the Local Health Insurance Funds (Allgemeine Ortskrankenkasse (AOK)), which was approved by the local institutional review board. The AOK is one of the largest German health insurers, accounting for more than 27 million (37%) statutory insured individuals in 2020. The statutory health insurance sector in turn covers nearly 90% of the 82 million German citizens [34].

The remuneration of health care services is regulated by mandatory coding rules, including the coding of diagnoses (International Statistical Classification of Diseases and Related Health Problems, German Modification; ICD-10 GM), procedures (German Procedure Classification; OPS) and drugs (Anatomical Therapeutic Chemical Classification System; ATC). For this study, all elderly patients (age at hospitalization ≥ 65 years) who had a coded diagnosis of PHF (ICD S42.2) and were treated with LPF (OPS 5-794.21 or 5-794.k1) from January 2010 to September 2018 were included for further analysis (*n* = 41,216). Patients with inconsistent data, previous humeral fracture fixation, both sides treated at index, polytrauma or bone cancer were excluded to guarantee a homogenous cohort.

**Figure 2:**
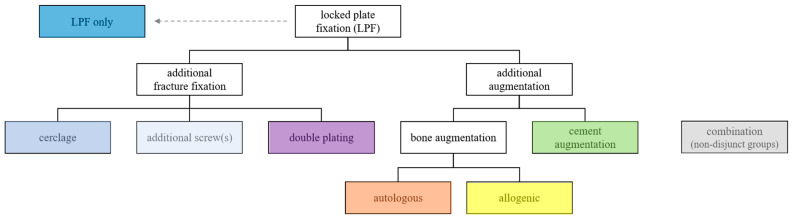
Treatment variants of locked plate fixation.

From the large variety of possible treatment options, we derived a systematic approach encompassing the most common, most discussed and readily codable treatment options to structure our further analysis, as depicted in Figure 2 below.

Baseline values were determined by diagnoses, procedures or medication in the two years prior to PHF. Co-morbidities were aggregated using the Charlson co-morbidity index (CCI; German modification) [35,36,37]. Outcomes were assessed by 30-day mortality, major adverse events (MAE; resuscitation, acute myocardial infarction, stroke, sepsis, acute renal failure, acute liver failure, acute respiratory distress syndrome, multiple organ failure or death); overall intra-hospital complication rate, infection, mechanical and non-mechanical surgical complications; and re-operation during hospitalization. Further and more detailed analysis was performed on sub-groups, which were formed according to the treatment options of locking plate fixation, as outlined in Figure 2: treated with LPF only; treated with additional fracture fixation including additional screw, cerclage or plate; treated with additional augmentation using cement or bone, including allogenic or autologous; or treated with a combination of these. The revenues of hospitals per case, which are equivalent to the reimbursements paid by health insurers, are reported as costs in this paper. The underlying codes are provided in the Supplementary Materials (Table S1) and were otherwise defined as in Köppe et al. [38].

Statistical analysis was performed to determine annual trends of use for each treatment variant and differences in age and sex distributions. Differences between treatment options were tested using two-sided Chi-squared and Kruskal–Wallis tests for categorical and continuous variables. All analyses were fully explorative, and *p*-values were thus understood in terms of hypothesis generation. *p*-values < 5% were interpreted as statistically noticeable. Information processing and analysis was performed using SAS software version 9.4 (SAS Institute Inc., Cary, NC, USA).

## 3. Results

### 3.1. Cohort and Treatment Sub-Groups

The analyzed cohort contains a total of 41,216 individuals, of which 32,952 (80%) were treated exclusively with a locking plate, while 5572 (14%) received additional fracture fixation, 1983 (5%) additional augmentation and 709 (2%) a combination of different materials. The coded numbers for each treatment variant are given in Table 1.

### 3.2. Trends over Time

**Figure 3:**
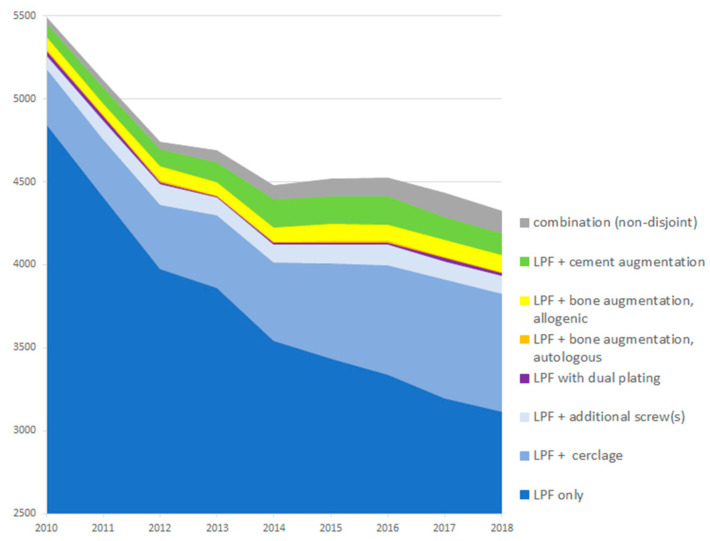
Trends of treatment variants over time. Due to the required follow-up period, data for the fourth quarter of 2018 were not included in the analysis. Therefore, values for 2018 were projected based on the first to third quarter. The figure was scaled so that values below 2500 were truncated.

As provided in the Supplementary Materials (Table S2) and visualized in Figure 3, the total number of treatments of PHFs with LPF steadily declined from 5487 in 2010 to around 4431 in 2017. In contrast, treatment variants with additional fracture fixation and/or augmentation increased in absolute and relative numbers, with the exception of the smallest sub-groups double plating (from 31 (1%) to 10 (0%)) and autologous bone augmentation (from 11 (0%) to 4 (0%)).

The relative change (based on the mean of the first/last three years) was −35% for LPF only, +58% for LPF + fracture fixation and +25% for LPF + augmentation.

### 3.3. Baseline Characteristics

Since some of the analyzed sub-groups are small, the following results describing the cohort and its baseline parameters are reported on an aggregated level in Table 2. The complete data are provided in the Supplementary Materials in Table S3.

The cohort had a median age of 78 years; 34,476 (84%) were women and 6740 (16%) men. Across all sub-groups, 12–14% of patients had a Charlson co-morbidity index of >5, as an indicator for a severe degree of co-morbidities. The aggregated prevalence across all sub-groups was 16,191 (39%) for diabetes, 10,262 (25%) for previous stroke, 7512 (18%) for atrial fibrillation and flutter, 12,070 (29%) for congestive heart failure, 12,471 (30%) for coronary heart disease, 35,830 (87%) for hypertension, 6815 (17%) for atherosclerosis and 10,857 (26%) for chronic kidney disease. Nicotine and alcohol abuse were notably more common in groups treated with additional variants (*p* < 0.001). Other complicating factors such as obesity, dementia or Parkinson’s disease showed no differences (Table 2). Surgically relevant injury of the axillary artery or brachial plexus was rare, with 25 and 151 cases overall, respectively (<1%). A total of 14,466 (35%) patients were diagnosed with osteoporosis, of which 4096 (10%) received an osteoporosis treatment. An osteoporosis diagnosis was notably more likely in the augmentation group, with 42% of cases, compared to an overall 35% (*p* < 0.001).

### 3.4. Outcomes and Complications

Outcomes and complications by sub-groups are shown in Table 3. The complete data are provided in the Supplementary Materials in Table S4.

Overall, intra-hospital death occurred in 675 cases (2%) and 30-day mortality in 908 cases (2%), with major adverse events in 1775 (4%) cases. The overall intra-hospital complication rate was 6211 (15%) and was highest in the augmentation sub-group (*p* < 0.001), with up to 22% (*n* = 17) in patients treated with additional autologous bone augmentation. Similarly, the re-operation rate of 3202 (8%) for the total population was notably higher in the augmentation sub-group (*n* = 213, 11%, *p* < 0.001). Mechanical complications were generally rare (<1%). Within surgical complications, non-mechanical complications lead, with 1082 (3%) ahead of mechanical complications with 233 (1%).

### 3.5. Length of Hospital Stay and Treatment Costs

Length of hospital stay (LOS) and treatment costs are shown in Table 4. The complete data are provided in the Supplementary Materials in Table S5.

The LOS differed between 14 and 16 days across sub-groups (*p* < 0.001). The incurred treatment costs increased from a mean EUR 6748.76 for LPF only to a maximum of EUR 8673.78 for a combination of different variants (*p* < 0.001).

## 4. Discussion

The most important findings of this study are that about 20% of the patients were treated with additional procedures to LPF. These treatments were associated with more complications, longer LOS and increased treatment costs.

Most patients were treated with LPF only. Additional treatment variants account for less than 20% of coded treatments, and excluding cerclages, less than 10%. Pre-selection of exclusively surgically treated elderly patients is accompanied by a clinically significant prevalence of osteoporosis and complex cases [39] because simple fractures are likely to be treated non-surgically and are consequently not included in this study. Given this combination as an indication for enhanced stabilization [40], a larger proportion of treatment variants from the augmentation sub-groups would be expected than the coded 5% for bone augmentation and cement augmentation combined. LPF with autologous bone augmentation was rarely used, which is probably due to the added complexity compared to allogenic bone grafts [29]. Similarly, LPF with double plating was used in less than one percent of the patients. It is possible that surgeons prefer arthroplasty in cases that typically would provide an indication for double plating in the elderly [24,41]. While there is an absolute and a substantial relative growth of additional fracture fixation and to a lesser extent augmentation, complementary materials are reserved for selected cases so far. Both double plating and autologous bone augmentation further decreased at an already low level and therefore seem to be reserved for special cases only.

The observed steady decline of LPF over the last decade is in line with literature describing a shift toward other treatment options [10,11,12]. Despite the small absolute number of additional treatment variants, trends over time and across variants do confirm the adoption of innovative developments. However, a comparison of the coded and imbursed number of cases with the literature and reported advances in surgical technique shows that the real world lags behind specialized trauma centers and the optimal treatment choices, as recommended in current reviews [42].

Patient characteristics and outcome for patients treated with LPF have been described in previous investigations [38,43,44]. A prevalence of 35% osteoporosis in this cohort lies at the far upper end of the expected range for the population [45]. When osteoporosis was present, augmentation was more frequently performed, as bio-mechanical properties would suggest. Notably, only 28% of patients diagnosed with osteoporosis received an osteoporosis treatment, unnecessarily increasing the risk of subsequent osteoporotic fractures, which was previously shown to be 36% after five years [43]. Risk factors for a reduced compliance differ regarding substance abuse, albeit with weak association. The largest difference was observed between the sub-groups variant combination and LPF only with 9% versus 5%, followed by 7% in additional augmentation and 6% in additional fracture fixation. We found no differences regarding dementia, which has previously been described as a risk factor [46]. Based on these results, surgeons should include risk factors—such as alcohol abuse—when making treatment decisions [47].

The outcomes show an association between more complex treatment variants and more frequent complications. In this study, significantly higher complication and re-operation rates were observed in the augmentation sub-groups. Since several studies had shown no increased complication rate after augmentation [48,49], a pre-selection of more complex cases that are necessarily being treated with bone or cement augmentation can be assumed. From a surgical point of view, surgical and especially mechanical complications were generally rare. The observed 30-day mortality of 2.2% in this treatment reality study is in the same order of magnitude as previously reported values [38] and consistent with 5.4%, as reported in the literature [7,50].

More complex treatment variants are associated with higher treatment costs, though at a similar LOS of roughly 15 days. The large variance of LOS and treatment costs is caused by the underlying exponential distribution and could be explained by the fact that a few patients with a very high LOS, e.g., because of an adverse course, pull the mean and, hence, the variance to higher values, which is typical for economic data.

This study has several limitations: Health claims data generally have an extraordinarily high external validity and can be considered a strong source of real-world evidence [51,52,53]. Although the analyzed data were not originally collected for research purposes, they are characterized particularly by their completeness, since uniform legal requirements exist for coding diagnoses and procedures using ICD and OPS and these codes are compulsory for reimbursement. Thus, the data capture the full range of the health care sector beyond specialized and highly publishing centers. Errors and aberrant coding practices among healthcare providers, if present, are likely to affect all groups uniformly. Nevertheless, it cannot be excluded that more complex procedures are actually performed but coded less frequently or completely (under-coding as a possible source of bias). As a limitation, fracture mechanism and exact patterns are unknown. Furthermore, there is no information on the size of hospitals or surgeons’ experience or treatment decision making, which might contribute to a selection bias. Potential bias would be reduced by the large amount of health claims data. No multi-variable analysis was performed in this predominantly descriptive study, since most sub-groups included only a small number of cases and showed no notable differences.

The objective of the study was to assess the implications of the presented real-world data. Further research on health technology assessment and cost-effectiveness must add to existing clinical studies in order to determine the advantageousness of specific treatment options from a socioeconomic perspective and to explain evolution of practice [54].

## 5. Conclusions

Based on real-world data of more than 40,000 patients, this study provides a broad assessment of the treatment reality of PHFs in elderly patients in Germany. It is consistent with the trend described in the literature of an overall decline of LPF in favor of alternative surgical procedures. At the same time, it confirms the adoption of innovative developments. There is both an absolute and relative increase in treatment variants, which collectively account for 20% of all coded plate fixations. This might indicate more personalized treatment pathways considering LPF’s limitations, complications and their prevention.

## Figures and Tables

**Table 1:** Treatment groups.

Procedure	*n* (%)
LPF only	32,952 (80%)
LPF + additional fracture fixation	5572 (14%)
-LPF + cerclage	4465 (11%)
-LPF + additional screw(s)	963 (2%)
-LPF with double plating	144 (0%)
LPF + additional augmentation	1983 (5%)
-LPF + bone augmentation, autologous	76 (0%)
-LPF + bone augmentation, allogenic	764 (2%)
-LPF + cement augmentation	1143 (3%)
combination (non-disjoint)	709 (2%)
Total	41,216 (100%)

Rounding error in LPF + fracture fixation group.

**Table 2:** Treatment groups. Description of the cohort and values at baseline.

	LPF Only	LPF + Fracture Fixation	LPF + Augmentation	Combination (Non-Disjoint)	*p*-Value
Age (median, IQR)	78 (11)	78 (11)	78 (11)	77 (11)	<0.001
Sex (women)	27,698 (84%)	4601 (83%)	1646 (83%)	531 (75%)	<0.001
Charlson co-morbidity index (median, IQR)	2 (3)	2 (3)	2 (3)	2 (3)	0.975
Obesity	8815 (27%)	1512 (27%)	551 (28%)	195 (28%)	0.778
Nicotine abuse	1923 (6%)	408 (7%)	119 (6%)	50 (7%)	<0.001
Alcohol abuse	1785 (5%)	309 (6%)	139 (7%)	61 (9%)	<0.001
Dementia	1768 (5%)	303 (5%)	95 (5%)	33 (5%)	0.571
Parkinson’s	1351 (4%)	208 (4%)	83 (4%)	26 (4%)	0.853
Seropositive chronic polyarthritis	1983 (6%)	338 (6%)	142 (7%)	42 (6%)	0.490
Arthritis of the shoulder	670 (2%)	131 (2%)	54 (3%)	18 (3%)	0.229
Frozen shoulder	1492 (5%)	223 (4%)	82 (4%)	41 (6%)	0.319
Rotator cuff tear	2012 (6%)	378 (7%)	165 (8%)	66 (9%)	<0.001
Osteoporosis	11,448 (35%)	1948 (35%)	824 (42%)	246 (35%)	<0.001
Osteoporosis treatment	3243 (10%)	561 (10%)	225 (11%)	67 (9%)	0.188

Absolute number (*n*=), interquartile range (IQR), percent of sub-group (%).

**Table 3:** Outcomes and complications.

	LPF Only	LPF + Fracture Fixation	LPF + Augmentation	Combination (Non-Disjoint)	*p*-Value
Overall intra-hospital complication rate	4915 (15%)	806 (14%)	374 (19%)	116 (16%)	<0.001
Infection	171 (1%)	39 (1%)	16 (1%)	5 (1%)	0.102
Non-mechanical surgical complication	855 (3%)	136 (2%)	69 (3%)	22 (3%)	0.070
Mechanical surgical complication	174 (1%)	32 (1%)	19 (1%)	8 (1%)	<0.001
Re-operation	2516 (8%)	408 (7%)	213 (11%)	65 (9%)	<0.001
Major adverse event	1388 (4%)	247 (4%)	108 (5%)	32 (5%)	0.108
30-day mortality	734 (2%)	115 (2%)	51 (3%)	8 (1%)	0.285

Absolute number (*n*=), percent of sub-group (%).

**Table 4:** Length of hospital stay and treatment costs.

	LPF Only	LPF + Fracture Fixation	LPF + Augmentation	Combination (Non-Disjoint)	*p*-Value
Length of stay (in days; mean, SD)	14.6 (11.4)	14.4 (11.2)	15.9 (12.2)	15.1 (12.0)	<0.001
Cost (in Euro; mean, SD)	6748.76 (±5626.63)	7987.40 (±4926.42)	8491.79 (±5087.06)	8673.78 (±5097.10)	<0.001

Standard deviation (SD).

## Data Availability

Restrictions apply to the availability of these data. Due to the Bundesdatenschutzgesetz (BDSG), the data used in this study cannot be made available in the manuscript, in the Supplementary Materials or in a public directory. Therefore, they are stored on a secure storage medium at the Wissenschaftliches Institut der AOK (WIdO) to facilitate replication of the results. Generally, access to data of statutory health insurance providers for research purposes is only possible under the conditions specified in the Sozialgesetzbuch (SGB V § 287). Requests for data access can be made as a formal request to the responsible data protection authority, specifying the recipient and the purpose of the data transfer. Access to the data used in this study can be granted to external parties only under the conditions of the cooperation agreement of this research project and after written approval by the health insurance company. For assistance in obtaining access to the data, please contact wido@wido.bv.aok.de.

## Supplementary Materials

The following supporting information can be downloaded at: https://www.mdpi.com/article/10.3390/jcm12041440/s1, Table S1: Sub-group coding for different treatment options; Table S2: Treatments per year by groups; Table S3: Description of the cohort and values at baseline (detailed); Table S4: Outcomes and complications (detailed); Table S5: Length of hospital stay and treatment costs (detailed).

## References

[1] Court-Brown C.M., Caesar B. (2006). Epidemiology of adult fractures: A review. Injury.

[2] Court-Brown C.M., Duckworth A.D., Clement N.D., McQueen M.M. (2018). Fractures in older adults. A view of the future?. Injury.

[3] Hemmann P., Ziegler P., Konrads C., Ellmerer A., Klopfer T., Schreiner A.J., Bahrs C. (2020). Trends in fracture development of the upper extremity in Germany-a population-based description of the past 15 years. J. Orthop. Surg. Res..

[4] Rupp M., Walter N., Pfeifer C., Lang S., Kerschbaum M., Krutsch W., Baumann F., Alt V. (2021). The Incidence of Fractures Among the Adult Population of Germany—And Analysis From 2009 through 2019. Dtsch. Arztebl. Int..

[5] Stolberg-Stolberg J., Köppe J., Rischen R., Freistühler M., Faldum A., Katthagen J.C., Raschke M.J. (2021). Einfluss von Komplikationen und Komorbiditäten auf Liegedauer und Kosten bei der operativen Behandlung der proximalen Humerusfraktur. Chirurg.

[6] Handoll H., Brealey S., Rangan A., Keding A., Corbacho B., Jefferson L., Chuang L.-H., Goodchild L., Hewitt C., Torgerson D. (2015). The ProFHER (PROximal Fracture of the Humerus: Evaluation by Randomisation) trial—A pragmatic multicentre randomised controlled trial evaluating the clinical effectiveness and cost-effectiveness of surgical compared with non-surgical treatment for proximal fracture of the humerus in adults. Health Technol. Assess..

[7] Lander S.T., Mahmood B., Maceroli M.A., Byrd J., Elfar J.C., Ketz J.P., Nikkel L.E. (2019). Mortality Rates of Humerus Fractures in the Elderly: Does Surgical Treatment Matter?. J. Orthop. Trauma.

[8] O’Donnell J.A., Gage M.J. (2021). Proximal Humerus Geriatric Fracture Care: Fix, Replace, or Nonoperative Treatment?. J. Orthop. Trauma.

[9] Bell J.-E., Leung B.C., Spratt K.F., Koval K.J., Weinstein J.D., Goodman D.C., Tosteson A.N.A. (2011). Trends and variation in incidence, surgical treatment, and repeat surgery of proximal humeral fractures in the elderly. J. Bone Jt. Surg. Am..

[10] Klug A., Gramlich Y., Wincheringer D., Schmidt-Horlohé K., Hoffmann R. (2019). Trends in surgical management of proximal humeral fractures in adults: A nationwide study of records in Germany from 2007 to 2016. Arch. Orthop. Trauma Surg..

[11] Patel A.H., Wilder J.H., Ofa S.A., Lee O.C., Savoie F.H., O’Brien M.J., Sherman W.F. (2022). Trending a decade of proximal humerus fracture management in older adults. JSES Int..

[12] Haasters F., Siebenbürger G., Helfen T., Daferner M., Böcker W., Ockert B. (2016). Complications of locked plating for proximal humeral fractures-are we getting any better?. J. Shoulder Elbow Surg..

[13] Barlow J.D., Logli A.L., Steinmann S.P., Sems S.A., Cross W.W., Yuan B.J., Torchia M.E., Sanchez-Sotelo J. (2020). Locking plate fixation of proximal humerus fractures in patients older than 60 years continues to be associated with a high complication rate. J. Shoulder Elbow Surg..

[14] Thanasas C., Kontakis G., Angoules A., Limb D., Giannoudis P. (2009). Treatment of proximal humerus fractures with locking plates: A systematic review. J. Shoulder Elbow Surg..

[15] Schliemann B., Siemoneit J., Theisen C., Kösters C., Weimann A., Raschke M.J. (2012). Complex fractures of the proximal humerus in the elderly—Outcome and complications after locking plate fixation. Musculoskelet. Surg..

[16] Schliemann B., Wähnert D., Theisen C., Herbort M., Kösters C., Raschke M.J., Weimann A. (2015). How to enhance the stability of locking plate fixation of proximal humerus fractures? An overview of current biomechanical and clinical data. Injury.

[17] Laux C.J., Grubhofer F., Werner C.M.L., Simmen H.-P., Osterhoff G. (2017). Current concepts in locking plate fixation of proximal humerus fractures. J. Orthop. Surg. Res..

[18] Nowak L.L., Dehghan N., McKee M.D., Schemitsch E.H. (2018). Plate fixation for management of humerus fractures. Injury.

[19] Sun Q., Wu X., Wang L., Cai M. (2020). The plate fixation strategy of complex proximal humeral fractures. Int. Orthop..

[20] Oppebøen S., Wikerøy A.K.B., Fuglesang H.F.S., Dolatowski F.C., Randsborg P.-H. (2018). Calcar screws and adequate reduction reduced the risk of fixation failure in proximal humeral fractures treated with a locking plate: 190 patients followed for a mean of 3 years. J. Orthop. Surg. Res..

[21] Newman J.M., Kahn M., Gruson K.I. (2015). Reducing Postoperative Fracture Displacement After Locked Plating of Proximal Humerus Fractures: Current Concepts. Am. J. Orthop..

[22] Stone M.A., Namdari S. (2019). Surgical Considerations in the Treatment of Osteoporotic Proximal Humerus Fractures. Orthop. Clin. N. Am..

[23] Choi S., Seo K.-B., Kwon Y.S., Kang H., Cho C., Rho J.Y. (2019). Dual plate for comminuted proximal humerus fractures. Acta Orthop. Belg..

[24] Warnhoff M., Jensen G., Dey Hazra R.-O., Theruvath P., Lill H., Ellwein A. (2021). Double plating—Surgical technique and good clinical results in complex and highly unstable proximal humeral fractures. Injury.

[25] Kim T., See C.W., Li X., Zhu D. (2020). Orthopedic implants and devices for bone fractures and defects: Past, present and perspective. Eng. Regen..

[26] Salinas A.J., Vallet-Regi M., Heikkilä J. (2018). Use of bioactive glasses as bone substitutes in orthopedics and traumatology. Bioactive Glasses.

[27] van Dam V., Trinh H.A., Rokaya D., Trinh D.H. (2022). Bone Augmentation for Implant Placement: Recent Advances. Int. J. Dent..

[28] Omid R., Trasolini N.A., Stone M.A., Namdari S. (2021). Principles of Locking Plate Fixation of Proximal Humerus Fractures. J. Am. Acad. Orthop. Surg..

[29] Biermann N., Prall W.C., Böcker W., Mayr H.O., Haasters F. (2019). Augmentation of plate osteosynthesis for proximal humeral fractures: A systematic review of current biomechanical and clinical studies. Arch. Orthop. Trauma Surg..

[30] Dasari S.P., Kerzner B., Fortier L.M., Rea P.M., Bodendorfer B.M., Chahla J., Garrigues G.E., Verma N.N. (2022). Improved outcomes for proximal humerus fracture open reduction internal fixation augmented with a fibular allograft in elderly patients: A systematic review and meta-analysis. J. Shoulder Elbow Surg..

[31] Sheng N., Wang Q., Chu G., Wang L., Cheng M., Weng Z., Wang Y., Rui B., Chen Y. (2021). Cancellous bone allograft is comparable to fibular strut allograft for augmentation in three- or four-part proximal humeral fractures. J. Shoulder Elbow Surg..

[32] Jabran A., Peach C., Ren L. (2018). Biomechanical analysis of plate systems for proximal humerus fractures: A systematic literature review. Biomed. Eng. Online.

[33] Foruria A.M., Martinez-Catalan N., Valencia M., Morcillo D., Calvo E. (2021). Proximal humeral fracture locking plate fixation with anatomic reduction, and a short-and-cemented-screws configuration, dramatically reduces the implant related failure rate in elderly patients. JSES Int..

[34] AOK-Bundesverband Zahlen und Fakten im Gesundheitswesen. https://aok-bv.de/hintergrund/zahlen-fakten/.

[35] Charlson M.E., Pompei P., Ales K.L., MacKenzie C. (1987). A new method of classifying prognostic comorbidity in longitudinal studies: Development and validation. J. Chronic Dis..

[36] Quan H., Li B., Couris C.M., Fushimi K., Graham P., Hider P., Januel J.-M., Sundararajan V. (2011). Updating and validating the Charlson comorbidity index and score for risk adjustment in hospital discharge abstracts using data from 6 countries. Am. J. Epidemiol..

[37] Stausberg J., Hagn S. (2015). New Morbidity and Comorbidity Scores based on the Structure of the ICD-10. PLoS ONE.

[38] Köppe J., Stolberg-Stolberg J., Rischen R., Faldum A., Raschke M.J., Katthagen J.C. (2021). In-hospital Complications Are More Likely to Occur After Reverse Shoulder Arthroplasty Than After Locked Plating for Proximal Humeral Fractures. Clin. Orthop. Relat. Res..

[39] Dey Hazra R.-O., Blach R.M., Ellwein A., Katthagen J.C., Lill H., Jensen G. (2022). Aktuelle Entwicklungen der Versorgungsrealität proximaler Humerusfrakturen—Eine Auswertung von 1162 Fällen an einem Level-1-Traumazentrum mit schulterchirurgischem Schwerpunkt. Z. Orthop. Unfall..

[40] Schuetze K., Eickhoff A., Röderer G., Gebhard F., Richter P.H. (2019). Osteoporotic Bone: When and How to Use Augmentation?. J. Orthop. Trauma.

[41] Katthagen J.C., Huber M., Grabowski S., Ellwein A., Jensen G., Lill H. (2017). Failure and revision rates of proximal humeral fracture treatment with the use of a standardized treatment algorithm at a level-1 trauma center. J. Orthop. Traumatol..

[42] Tepass A., Weise K., Rolauffs B., Blumenstock G., Bahrs C. (2015). Behandlung proximaler Humerusfrakturen in Deutschland: Einfluss von Krankenhausversorgungsstufe und Behandlungsfrequenz: [Treatment of proximal humeral fractures in Germany: Influence of the level of hospital care and the frequency of treatment]. Unfallchirurg.

[43] Stolberg-Stolberg J., Köppe J., Rischen R., Freistühler M., Faldum A., Katthagen J.C., Raschke M.J. (2021). The Surgical Treatment of Proximal Humeral Fractures in Elderly Patients. Dtsch. Arztebl. Int..

[44] Koeppe J., Katthagen J.C., Rischen R., Freistuehler M., Faldum A., Raschke M.J., Stolberg-Stolberg J. (2021). Male Sex Is Associated with Higher Mortality and Increased Risk for Complications after Surgical Treatment of Proximal Humeral Fractures. J. Clin. Med..

[45] Hadji P., Klein S., Gothe H., Häussler B., Kless T., Schmidt T., Steinle T., Verheyen F., Linder R. (2013). The epidemiology of osteoporosis—Bone Evaluation Study (BEST): An analysis of routine health insurance data. Dtsch. Arztebl. Int..

[46] Mosk C.A., Mus M., Vroemen J.P., van der Ploeg T., Vos D.I., Elmans L.H., van der Laan L. (2017). Dementia and delirium, the outcomes in elderly hip fracture patients. Clin. Interv. Aging.

[47] Neuhaus V., Swellengrebel C.H.J., Bossen J.K.J., Ring D. (2013). What are the factors influencing outcome among patients admitted to a hospital with a proximal humeral fracture?. Clin. Orthop. Relat. Res..

[48] Kim D.-S., Lee D.-H., Chun Y.-M., Shin S.-J. (2018). Which additional augmented fixation procedure decreases surgical failure after proximal humeral fracture with medial comminution: Fibular allograft or inferomedial screws?. J. Shoulder Elbow Surg..

[49] Hengg C., Nijs S., Klopfer T., Jaeger M., Platz A., Pohlemann T., Babst R., Franke J., Kralinger F. (2019). Cement augmentation of the proximal humerus internal locking system in elderly patients: A multicenter randomized controlled trial. Arch. Orthop. Trauma Surg..

[50] Robinson C.M., Stirling P.H.C., Goudie E.B., MacDonald D.J., Strelzow J.A. (2019). Complications and Long-Term Outcomes of Open Reduction and Plate Fixation of Proximal Humeral Fractures. J. Bone Jt. Surg. Am..

[51] Blonde L., Khunti K., Harris S.B., Meizinger C., Skolnik N.S. (2018). Interpretation and Impact of Real-World Clinical Data for the Practicing Clinician. Adv. Ther..

[52] Stausberg J., Lang H., Obertacke U., Rauhut F. (2001). Classifications in routine use: Lessons from ICD-9 and ICPM in surgical practice. J. Am. Med. Inform. Assoc..

[53] Behrendt C.-A., Debus E.S., Mani K., Sedrakyan A. (2018). The Strengths and Limitations of Claims Based Research in Countries With Fee for Service Reimbursement. Eur. J. Vasc. Endovasc. Surg..

[54] Mellstrand Navarro C., Brolund A., Ekholm C., Heintz E., Hoxha Ekström E., Josefsson P.O., Leander L., Nordström P., Zidén L., Stenström K. (2018). Treatment of humerus fractures in the elderly: A systematic review covering effectiveness, safety, economic aspects and evolution of practice. PLoS ONE.

