# Supplementary material for: Treatment Reality of Proximal Humeral Fractures in the Elderly—Trending Variants of Locking Plate Fixation in Germany
Source: J Clin Med. 2023 Feb 10;12(4):1440. doi: 10.3390/jcm12041440 (PMC9963188; doi:10.3390/jcm12041440)
Supplement: Supplementary file 1 [file jcm-12-01440-s001.zip › jcm-2182327-supplementary.pdf]

Supplementary Materials

Table S1. Subgroup coding for different treatment options.

Treatment Option	OPS-Code
LPF only	5-794.21, 5-794.k1
LPF + cerclage	5-793.21, 5-794.11, 5-794.f1, 5-78a.11
LPF + additional screw(s)	5-793.11, 5-794.01, 5-78a.01, 5-786.0
LPF with double plating	5-793.31, 5-793.k1, 5-794.21, 5-794.k1 if coded twice or with both codes for regular and locking pate
LPF + bone augmentation, autologous	5-784.01, 5-784.11, 5-784.21, 5-784.31, 5-784.41, 5-784.51
LPF + bone augmentation, allogenic	5-784.61, 5-784.71, 5-784.81
LPF + cement augmentation	5-785.01, 5-785.11, 5-86.a3

Table S2. Treatments per year by groups.

Treatments per Year	LPF Only	LPF + Fracture Fixation	LPF + Cerclage	LPF + Additional Screw(s)	LPF with Double Plating	LPF + Augmentation	LPF + Bone Augmentation, Autologous	LPF + Bone Augmentation, Allogenic	LPF + Cement Augmentation	Combination (Non-Disjoint)	Total
**2010**	4847 (88%)	446 (8%)	335 (6%)	80 (1%)	31 (1%)	164 (3%)	11 (0%)	74 (1%)	79 (1%)	30 (1%)	5487
**2011**	4408 (86%)	489 (10%)	350 (7%)	114 (2%)	25 (0%)	172 (3%)	8 (0%)	66 (1%)	98 (2%)	41 (1%)	5110
**2012**	3978 (84%)	524 (11%)	388 (8%)	124 (3%)	12 (0%)	198 (4%)	8 (0%)	87 (2%)	103 (2%)	40 (1%)	4740
**2013**	3864 (82%)	551 (12%)	434 (9%)	112 (2%)	5 (0%)	206 (4%)	8 (0%)	77 (2%)	121 (3%)	68 (1%)	4689
**2014**	3546 (79%)	590 (13%)	471 (11%)	106 (2%)	13 (0%)	259 (6%)	8 (0%)	82 (2%)	169 (4%)	83 (2%)	4478
**2015**	3435 (76%)	703 (16%)	575 (13%)	115 (3%)	13 (0%)	277 (6%)	10 (0%)	102 (2%)	165 (4%)	101 (2%)	4516
**2016**	3338 (74%)	799 (18%)	664 (15%)	122 (3%)	13 (0%)	280 (6%)	10 (0%)	94 (2%)	176 (4%)	106 (2%)	4523
**2017**	3196 (72%)	846 (19%)	717 (16%)	107 (2%)	22 (0%)	247 (6%)	9 (0%)	102 (2%)	136 (3%)	142 (3%)	4431
**2018 (Q1–Q3)**	2340 (72%)	624 (19%)	531 (16%)	83 (3%)	10 (0%)	180 (6%)	4 (0%)	80 (2%)	96 (3%)	98 (3%)	3242

Table S3. Description of the cohort and values at baseline (detailed).

	LPF Only	LPF + Fracture Fixation	LPF + Cerclage	LPF + Additional Screw(s)	LPF with Double Plating	LPF + Augmentation	LPF + Bone Augmentation, Autologous	LPF + Bone Augmentation, Allogenic	LPF + Cement Augmentation	Combination (Non-Disjoint)	Total	p-Value
Age (median, iqr)	78 (±11)	78 (±11)	78 (±10)	77 (±10)	79 (±11)	78 (±11)	76 (±12)	78 (±10)	79 (±10)	77 (±11)	78 (±11)	<.0001
Sex (women)	27,698 (84%)	4601 (83%)	3727 (83%)	750 (78%)	124 (86%)	1646 (83%)	57 (75%)	619 (81%)	970 (85%)	531 (75%)	34,476 (84%)	<.0001
Charlson comorbidity index (median, iqr)	2 (±3)	2 (±3)	2 (±3)	2 (±3)	2 (±3)	2 (±3)	2 (±3)	3 (±3)	2 (±3)	2 (±3)	2 (±3)	0.975
Obesity	8815 (27%)	1512 (27%)	1199 (27%)	278 (29%)	35 (24%)	551 (28%)	19 (25%)	215 (28%)	317 (28%)	195 (28%)	11,073 (27%)	0.7775

Nicotine abuse	1923 (6%)	408 (7%)	311 (7%)	85 (9%)	12 (8%)	119 (6%)	5 (7%)	38 (5%)	76 (7%)	50 (7%)	2500 (6%)	0.0003
Alcohol abuse	1785 (5%)	309 (6%)	245 (5%)	57 (6%)	7 (5%)	139 (7%)	8 (11%)	48 (6%)	83 (7%)	61 (9%)	2294 (6%)	0.0008
Diabetes	13,009 (39%)	2117 (38%)	1677 (38%)	384 (40%)	56 (39%)	791 (40%)	23 (30%)	318 (42%)	450 (39%)	274 (39%)	16,191 (39%)	0.1515
Previous stroke	8185 (25%)	1414 (25%)	1132 (25%)	251 (26%)	31 (22%)	497 (25%)	19 (25%)	193 (25%)	285 (25%)	166 (23%)	10,262 (25%)	0.883
Atrial fibrillation and flutter	6013 (18%)	996 (18%)	799 (18%)	166 (17%)	31 (22%)	371 (19%)	15 (20%)	146 (19%)	210 (18%)	132 (19%)	7512 (18%)	0.917
Congestive heart failure	9790 (30%)	1543 (28%)	1248 (28%)	250 (26%)	45 (31%)	557 (28%)	16 (21%)	228 (30%)	313 (27%)	180 (25%)	12,070 (29%)	0.0026
Coronary heart disease	10,053 (31%)	1625 (29%)	1303 (29%)	281 (29%)	41 (28%)	595 (30%)	25 (33%)	226 (30%)	344 (30%)	198 (28%)	12,471 (30%)	0.4907
Hypertension	28,685 (87%)	4797 (86%)	3814 (85%)	858 (89%)	125 (87%)	1736 (88%)	61 (80%)	673 (88%)	1002 (88%)	612 (86%)	35,830 (87%)	0.0119
Atherosclerosis	5335 (16%)	980 (18%)	806 (18%)	155 (16%)	19 (13%)	381 (19%)	8 (11%)	158 (21%)	215 (19%)	119 (17%)	6815 (17%)	0.0003
Chronic kidney disease	8653 (26%)	1451 (26%)	1158 (26%)	257 (27%)	36 (25%)	566 (29%)	13 (17%)	241 (32%)	312 (27%)	187 (26%)	10,857 (26%)	0.0332
Cancer	7123 (22%)	1273 (23%)	1034 (23%)	215 (22%)	24 (17%)	436 (22%)	15 (20%)	167 (22%)	254 (22%)	164 (23%)	8996 (22%)	0.2609
Dementia	1768 (5%)	303 (5%)	244 (5%)	47 (5%)	12 (8%)	95 (5%)	2 (3%)	38 (5%)	55 (5%)	33 (5%)	2199 (5%)	0.5713
Parkinson's	1351 (4%)	208 (4%)	165 (4%)	37 (4%)	6 (4%)	83 (4%)	2 (3%)	36 (5%)	45 (4%)	26 (4%)	1668 (4%)	0.8529
Seropositive chronic polyarthritis	1983 (6%)	338 (6%)	270 (6%)	60 (6%)	8 (6%)	142 (7%)	4 (5%)	49 (6%)	89 (8%)	42 (6%)	2505 (6%)	0.4896
Arthritis of the shoulder	670 (2%)	131 (2%)	110 (2%)	19 (2%)	2 (1%)	54 (3%)	2 (3%)	24 (3%)	28 (2%)	18 (3%)	873 (2%)	0.2286
Frozen shoulder	1492 (5%)	223 (4%)	177 (4%)	39 (4%)	7 (5%)	82 (4%)	4 (5%)	36 (5%)	42 (4%)	41 (6%)	1838 (4%)	0.3185
Rotator cuff tear	2012 (6%)	378 (7%)	302 (7%)	69 (7%)	7 (5%)	165 (8%)	9 (12%)	69 (9%)	87 (8%)	66 (9%)	2621 (6%)	<.0001
Injury of axillary artery	16 (0%)	5 (0%)	4 (0%)	0 (0%)	1 (1%)	3 (0%)	0 (0%)	1 (0%)	2 (0%)	1 (0%)	25 (0%)	0.0309
Brachial plexus injury	116 (0%)	13 (0%)	11 (0%)	2 (0%)	0 (0%)	13 (1%)	0 (0%)	4 (1%)	9 (1%)	9 (1%)	151 (0%)	0.0007
Previous shoulder surgery	74 (0%)	14 (0%)	10 (0%)	4 (0%)	0 (0%)	9 (0%)	1 (1%)	4 (1%)	4 (0%)	2 (0%)	99 (0%)	0.2658
Osteoporosis	11,448 (35%)	1948 (35%)	1590 (36%)	307 (32%)	51 (35%)	824 (42%)	40 (53%)	301 (39%)	483 (42%)	246 (35%)	14,466 (35%)	<.0001
Osteoporosis treatment	3243 (10%)	561 (10%)	462 (10%)	87 (9%)	12 (8%)	225 (11%)	13 (17%)	84 (11%)	128 (11%)	67 (9%)	4096 (10%)	0.188

Absolute number (n=), standard deviation (SD), percent (%). Note that discrepancies between fine granular level and aggregated categories arise from different amounts of non-disjoint groups for each level.

Table S4. Outcomes and complications (detailed).

	LPF Only	LPF + Fracture Fixation	LPF + Cerclage	LPF + Additional Screw(s)	LPF with Double Plating	LPF + Augmentation	LPF + Bone Augmentation, Autologous	LPF + Bone Augmentation, Allogenic	LPF + Cement Augmentation	Combination (Non-Disjoint)	Total	p-Value
Overall intrahospital complication rate	4915 (15%)	806 (14%)	633 (14%)	150 (16%)	23 (16%)	374 (19%)	17 (22%)	148 (19%)	209 (18%)	116 (16%)	6211 (15%)	0.0002
Consequence of injury	47 (0%)	9 (0%)	6 (0%)	2 (0%)	1 (1%)	6 (0%)	1 (1%)	3 (0%)	2 (0%)	1 (0%)	63 (0%)	0.0736
Implant malposition	39 (0%)	8 (0%)	7 (0%)	0 (0%)	1 (1%)	5 (0%)	0 (0%)	3 (0%)	2 (0%)	4 (1%)	56 (0%)	0.0081
Mechanical complication (dysfuntion/failure)	43 (0%)	9 (0%)	8 (0%)	0 (0%)	1 (1%)	6 (0%)	0 (0%)	3 (0%)	3 (0%)	4 (1%)	62 (0%)	0.0142
Mechanical complication (loosening)	39 (0%)	8 (0%)	7 (0%)	0 (0%)	1 (1%)	5 (0%)	0 (0%)	3 (0%)	2 (0%)	4 (1%)	56 (0%)	0.0081
Periprosthetic fractures	46 (0%)	12 (0%)	9 (0%)	3 (0%)	0 (0%)	6 (0%)	0 (0%)	3 (0%)	3 (0%)	3 (0%)	67 (0%)	0.2301
Peri-implant fracture	77 (0%)	18 (0%)	14 (0%)	3 (0%)	1 (1%)	11 (1%)	0 (0%)	6 (1%)	5 (0%)	6 (1%)	112 (0%)	0.0046
Luxation	1011 (3%)	247 (4%)	197 (4%)	47 (5%)	3 (2%)	91 (5%)	2 (3%)	41 (5%)	48 (4%)	44 (6%)	1393 (3%)	<.0001
Necrosis	72 (0%)	10 (0%)	8 (0%)	2 (0%)	0 (0%)	9 (0%)	0 (0%)	4 (1%)	5 (0%)	0 (0%)	91 (0%)	0.3294
Nerve injury	74 (0%)	8 (0%)	5 (0%)	2 (0%)	1 (1%)	12 (1%)	0 (0%)	4 (1%)	8 (1%)	4 (1%)	98 (0%)	0.0041
Vascular injury	20 (0%)	5 (0%)	4 (0%)	0 (0%)	1 (1%)	5 (0%)	0 (0%)	2 (0%)	3 (0%)	1 (0%)	31 (0%)	0.0101
Haematoma	715 (2%)	102 (2%)	83 (2%)	16 (2%)	3 (2%)	57 (3%)	3 (4%)	18 (2%)	36 (3%)	18 (3%)	892 (2%)	0.1824
Compartment syndrome	199 (1%)	51 (1%)	36 (1%)	14 (1%)	1 (1%)	16 (1%)	0 (0%)	4 (1%)	12 (1%)	7 (1%)	273 (1%)	0.019
Impingement	67 (0%)	3 (0%)	2 (0%)	1 (0%)	0 (0%)	6 (0%)	2 (3%)	1 (0%)	3 (0%)	1 (0%)	77 (0%)	<.0001
Bursitis	27 (0%)	9 (0%)	8 (0%)	0 (0%)	1 (1%)	1 (0%)	1 (1%)	0 (0%)	0 (0%)	1 (0%)	38 (0%)	0.0008
Infection	171 (1%)	39 (1%)	34 (1%)	4 (0%)	1 (1%)	16 (1%)	2 (3%)	5 (1%)	9 (1%)	5 (1%)	231 (1%)	0.1019
Infection with antibiotic resistance	34 (0%)	2 (0%)	1 (0%)	0 (0%)	1 (1%)	3 (0%)	1 (1%)	1 (0%)	1 (0%)	2 (0%)	41 (0%)	0.002
Sepsis	156 (0%)	19 (0%)	11 (0%)	7 (1%)	1 (1%)	15 (1%)	1 (1%)	7 (1%)	7 (1%)	6 (1%)	196 (0%)	0.0681
Non-mechanical surgical complication	855 (3%)	136 (2%)	114 (3%)	19 (2%)	3 (2%)	69 (3%)	5 (7%)	21 (3%)	43 (4%)	22 (3%)	1082 (3%)	0.0701
Mechanical surgical complication	174 (1%)	32 (1%)	26 (1%)	4 (0%)	2 (1%)	19 (1%)	3 (4%)	7 (1%)	9 (1%)	8 (1%)	233 (1%)	0.0007
Surgical incident	130 (0%)	23 (0%)	14 (0%)	6 (1%)	3 (2%)	18 (1%)	0 (0%)	6 (1%)	12 (1%)	4 (1%)	175 (0%)	0.0005
Re-operation	2516 (8%)	408 (7%)	318 (7%)	76 (8%)	14 (10%)	213 (11%)	11 (14%)	78 (10%)	124 (11%)	65 (9%)	3202 (8%)	<.0001
Additional surgery	1457 (4%)	222 (4%)	177 (4%)	39 (4%)	6 (4%)	111 (6%)	3 (4%)	46 (6%)	62 (5%)	38 (5%)	1828 (4%)	0.1238

Blood transfusion	4877 (15%)	812 (15%)	631 (14%)	154 (16%)	27 (19%)	379 (19%)	18 (24%)	142 (19%)	219 (19%)	121 (17%)	6189 (15%)	<.0001
Thromboembolism	316 (1%)	52 (1%)	40 (1%)	11 (1%)	1 (1%)	22 (1%)	2 (3%)	11 (1%)	9 (1%)	12 (2%)	402 (1%)	0.2645
Deep vein thrombosis	56 (0%)	13 (0%)	10 (0%)	3 (0%)	0 (0%)	1 (0%)	0 (0%)	0 (0%)	1 (0%)	2 (0%)	72 (0%)	0.7342
Pulmonary embolism	84 (0%)	18 (0%)	11 (0%)	6 (1%)	1 (1%)	8 (0%)	1 (1%)	4 (1%)	3 (0%)	2 (0%)	112 (0%)	0.1553
Ards	7 (0%)	4 (0%)	3 (0%)	1 (0%)	0 (0%)	1 (0%)	0 (0%)	0 (0%)	1 (0%)	1 (0%)	13 (0%)	0.2791
Stroke	235 (1%)	39 (1%)	34 (1%)	5 (1%)	0 (0%)	16 (1%)	2 (3%)	7 (1%)	7 (1%)	8 (1%)	298 (1%)	0.3492
Acute myocardial infarction	209 (1%)	28 (1%)	26 (1%)	2 (0%)	0 (0%)	12 (1%)	0 (0%)	6 (1%)	6 (1%)	1 (0%)	250 (1%)	0.3858
Acute renal failure	558 (2%)	119 (2%)	98 (2%)	19 (2%)	2 (1%)	49 (2%)	3 (4%)	19 (2%)	27 (2%)	17 (2%)	743 (2%)	0.0542
Acute liver failure	35 (0%)	9 (0%)	8 (0%)	0 (0%)	1 (1%)	2 (0%)	0 (0%)	2 (0%)	0 (0%)	1 (0%)	47 (0%)	0.183
Multiple organ failure	0 (0%)	0 (0%)	0 (0%)	0 (0%)	0 (0%)	0 (0%)	0 (0%)	0 (0%)	0 (0%)	0 (0%)	0 (0%)	./.
Delirium	1317 (4%)	211 (4%)	170 (4%)	36 (4%)	5 (3%)	88 (4%)	3 (4%)	41 (5%)	44 (4%)	27 (4%)	1643 (4%)	0.7142
Reanimation	135 (0%)	21 (0%)	18 (0%)	2 (0%)	1 (1%)	8 (0%)	0 (0%)	6 (1%)	2 (0%)	2 (0%)	166 (0%)	0.5294
Intensive care treatment	899 (3%)	120 (2%)	86 (2%)	32 (3%)	2 (1%)	57 (3%)	4 (5%)	22 (3%)	31 (3%)	22 (3%)	1098 (3%)	0.0351
General comlication	2924 (9%)	475 (9%)	375 (8%)	90 (9%)	10 (7%)	203 (10%)	8 (11%)	90 (12%)	105 (9%)	66 (9%)	3668 (9%)	0.1544
Major adverse event	1388 (4%)	247 (4%)	202 (5%)	41 (4%)	4 (3%)	108 (5%)	5 (7%)	49 (6%)	54 (5%)	32 (5%)	1775 (4%)	0.1078
Intra-hospital death	544 (2%)	83 (1%)	63 (1%)	17 (2%)	3 (2%)	41 (2%)	0 (0%)	24 (3%)	17 (1%)	7 (1%)	675 (2%)	0.0275
30-day mortality	734 (2%)	115 (2%)	92 (2%)	20 (2%)	3 (2%)	51 (3%)	2 (3%)	25 (3%)	24 (2%)	8 (1%)	908 (2%)	0.2851

Table S5. Length of hospital stay and treatment costs (detailed).

	LPF Only	LPF + Fracture Fixation	LPF + Cerclage	LPF + Additional Screw(s)	LPF with Double Plating	LPF + Augmentation	LPF + Bone Augmentation, Autologous	LPF + Bone Augmentation, Allogenic	LPF + Cement Augmentation	Combination (Non-Disjoint)
Length of stay (in days; mean, SD)	14.6 (±11.4)	14.4 (±11.2)	14.2 (±10.9)	14.6 (±10.7)	17.4 (±15.1)	15.9 (±12.2)	15.1 (±11.1)	16.2 (±12.7)	15.6 (±11.7)	15.1 (±12.0)
Cost (in Euro; mean, SD)	6748.76 (±5626.63)	7987.40 (±4926.42)	7981.70 (±4963.54)	7770.88 (±4311.32)	8843.29 (±5196.16)	8491.79 (±5087.06)	8531.74 (±2892.88)	8822.72 (±5372.02)	8247.19 (±5006.28)	8673.78 (±5097.10)
